# Supplementary material for: Molecular Basis of a Dominant SARS-CoV-2 Spike-Derived Epitope Presented by HLA-A*02:01 Recognised by a Public TCR
Source: Cells. 2021 Oct 3;10(10):2646. doi: 10.3390/cells10102646 (PMC8534114; doi:10.3390/cells10102646)
Supplement: Supplementary file 1 [file cells-10-02646-s001.zip › cells-1362076-supplementary.pdf]

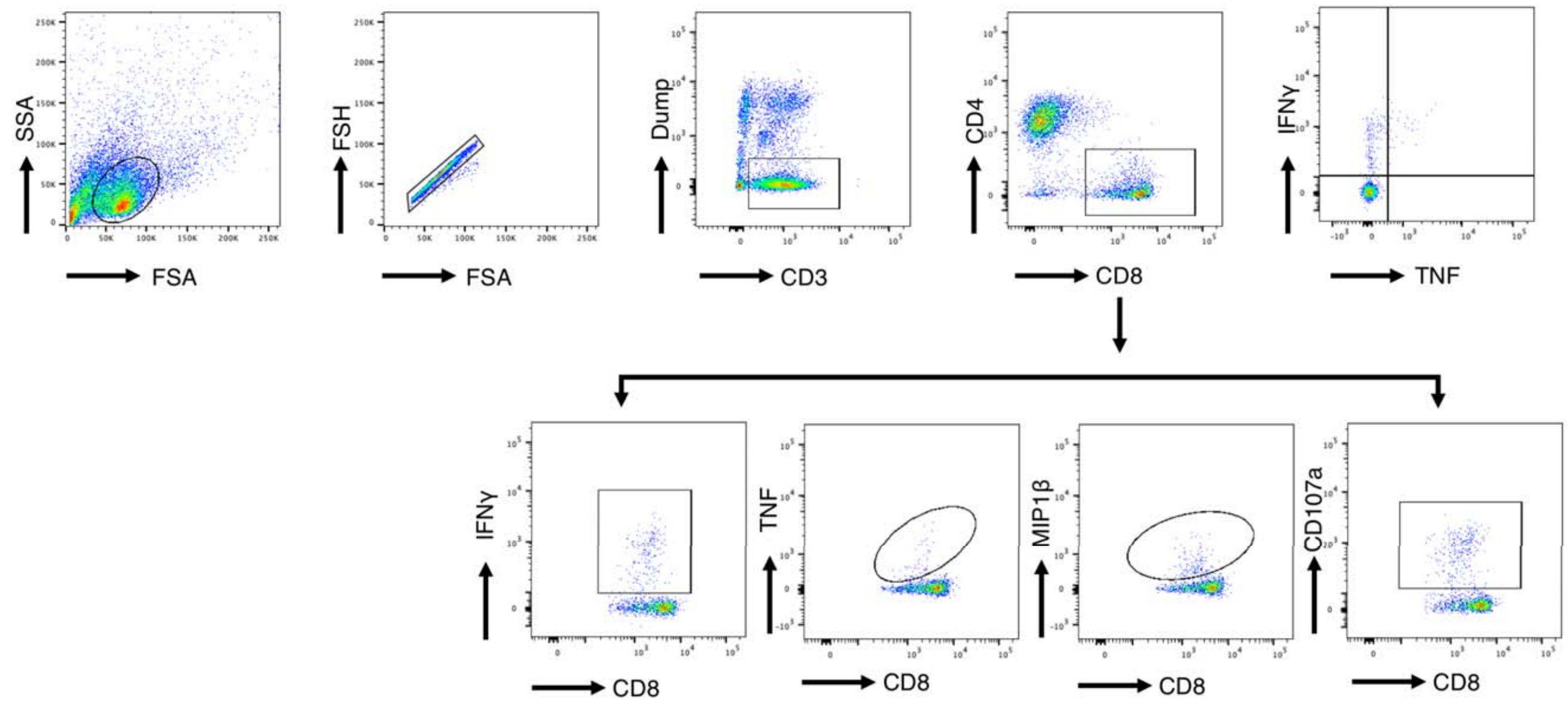

Figure S1. Gating strategy used in this study. (**Top**) Gating strategy used to assess the functional responses of CD8+ T cell lines. Cells were gated on lymphocytes, singlets, CD3+Live cells, CD8+CD4- T cells to observe IFNγ and TNF production. (**Bottom**) Gating strategy used to assess the polyfunctional responses of CD8+ T cell lines. Cells were gated on lymphocytes, singlets, CD3+Live cells, CD8+CD4- T cells and each of CD8+IFNγ+, CD8+TNF+, CD8+MIP1β and CD8+CD107a.
